# Using patient and physician perspectives to develop a shared decision-making framework for colorectal cancer

**DOI:** 10.1186/1748-5908-4-81

**Published:** 2009-12-24

**Authors:** Marisa Leon-Carlyle, Gillian Spiegle, Selina Schmocker, Anna Gagliardi, David Urbach, Erin Kennedy

**Affiliations:** 1 Department of Surgery, Toronto General Hospital, Toronto, Ontario, Canada; 2 Department of Health Policy, Management and Evaluation, University of Toronto, Toronto, Ontario, Canada

## Abstract

**Background:**

Colorectal cancer is the third leading cause of death from cancer worldwide with over 900,000 diagnoses and 639,000 deaths each year. Although shared decision making is broadly advocated as a mechanism by which to achieve patient-centred care, there has been little investigation of patient and physician shared decision-making preferences and practices or the outcomes associated with shared decision making in the context of colorectal cancer.

**Aim:**

The aim of this study is to determine patient and physician attitudes towards the use of shared decision making in the setting of colorectal cancer.

**Methods:**

Standard principles of qualitative research will be used to sample and interview 20 colorectal cancer patients in each of three tertiary care hospitals (n = 60) and 15 surgeons, radiation oncologists, and medical oncologists (n = 45) affiliated with cancer centres. The interview questions will be guided by a conceptual framework defining patient and physician factors that influence the shared decision-making process and associated outcomes in the setting of colorectal cancer. An inductive, grounded approach will be used by two investigators to independently analyze the interview transcripts. These investigators will meet to compare and achieve consensus on themes that will be tabulated to compare barriers, enablers, and outcomes of shared decision making by patient, physician, and contextual factors.

**Discussion:**

This study is the first to examine both patient and physician perspectives on the use of shared decision making for colorectal cancer in North America or elsewhere. It will provide a framework that can be used to describe the shared decision-making process and its outcomes, and evaluate strategies to facilitate this process for patients with colorectal cancer.

## Background

Colorectal cancer is the third leading cause of death from cancer worldwide, affecting 639,000 men and women annually [1]. The mainstay of treatment for colon cancer is surgery [2]. Following surgery, adjuvant chemotherapy is recommended for patients with stage three and stage four colon cancer, and follow-up for all stages is continued for approximately five years [2]. Patients with early rectal cancer (stage one) follow the same treatment algorithm as for colon cancer, while patients with stage two and three rectal cancer usually receive pre-operative chemoradiation for five weeks, followed by surgery six to eight weeks after the completion of their chemoradiation, and then receive post-operative chemotherapy [2]. Similar to colon cancer, follow-up for all stages of rectal cancer is continued for approximately five years. Following treatment for colorectal cancer, patients will experience long-term changes in bowel function that include increased number of bowel movements and urgency, as well as noticeable changes in sexual and bladder function [3-6]. These are profoundly important issues that must be considered by both the patient and the surgeon in order to plan treatment that is most consistent with the patient's values and lifestyle. Recently, several national research bodies have identified patient-centred care as a priority research theme [7,8]. Patient-centred care is defined as a 'collaboration between informed, respected patients and their families and a coordinated healthcare team to achieve quality healthcare' with the focus on the participation and engagement of the patient. Although several models of the patient-physician encounter have been described, shared decision making is broadly advocated as a mechanism by which to achieve patient-centred care [9,10] and has been shown to significantly increase patient knowledge, improve patient satisfaction with medical care and quality of life, and reduce anxiety and decisional conflict [11,12]. Shared decision making is characterized by a two-way flow of information during which the physician shares technical information (diagnosis, treatment alternatives, risks, benefits, outcomes), and the patient shares personal information during the encounter (lifestyle, work and family responsibilities, relationships, beliefs, fears) that the physician has no way of knowing except through direct communication with the patient [9,10]. Following this, both the patient and physician work together towards a final treatment decision.

Although most patients prefer an active or shared role in treatment decision making, their preferred roles are often not achieved [13-15]. Of 233 cancer patients attending outpatient clinic, 63% preferred an active role or shared role in decision making, but only 34% achieved this role [13,14]. In addition, patients who achieved a shared role in treatment decision making were more satisfied with the consultation and the information and emotional support received by their physician compared to those who achieved passive or active roles [13,16]. Despite surveys reporting that physicians are aware of what shared decision making is and have positive attitudes towards its use, implementation into clinical practice remains challenging [17-21]. The reasons for this seem to be lack of time, resources, and suitability of the decision aid for individual patients [21,22].

## Aims

The aims of this study are: to explore patients' and physicians' attitudes towards the use of shared decision making in the setting of colorectal cancer; to identify and explore the enablers and barriers to the use of shared decision making in the setting of colorectal cancer; and identify and explore strategies to promote shared decision making in the setting of colorectal cancer.

## Methods

### Overview

Prior to the start of this project, ethics approval will be obtained. Standard principles of qualitative research methods will be used to conduct interviews with colorectal cancer patients and their physicians to learn whether and how shared decision making is taking place, the factors influencing this process, associated outcomes, and suggestions for improving shared decision making for colorectal cancer.

### Conceptual framework

**Figure 1:**
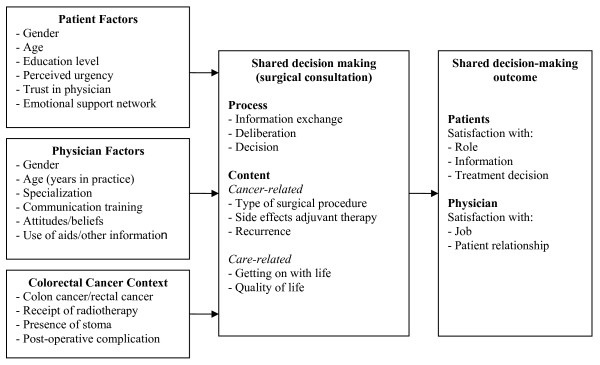
**Conceptual framework for shared decision making during the surgical consultation**.

Despite the considerable challenges and trade-offs associated with decisions for colorectal cancer, there has been little investigation into the shared decision-making preferences, practices, or outcomes associated with patients and physicians in the setting of colorectal cancer in Canada or elsewhere. We interviewed Ontario colorectal cancer patients who varied by age, gender, and stage of care, and found that that involvement in treatment decision making was considered very important by all patients [23]. Others studying the decisional support needs of patients with colorectal cancer have found that patient needs for information are high, and younger, female patients were more likely to prefer an active role in treatment decision making [24,25]. In addition, trust in the physician, having an emotional support network, and the perception that the patient's condition and treatment was considered important by the healthcare team also influenced colorectal cancer patients desire for involvement in treatment decision making, and was considered as important as the cancer information they received [24,25]. To date, no existing model of shared decision making incorporates the multiple factors that can influence this practice, the various elements of shared decision making or associated outcomes that have been discussed in the context of colorectal cancer. Therefore, we have combined these factors within the following conceptual framework (Figure 1) that will be used to define our research questions, approach, and data collection tools.

### Sampling and recruitment

Interviews will be conducted with 20 patients in each of three tertiary care teaching hospitals in a single large urban city, for a total of 60 interviews. These sites were chosen because they offer access to a high volume of colorectal cancer patients and because they maintain a clinical database that prospectively follow all colorectal cancer patients by which patients can be identified. Using these databases, patients will be selected that vary in non-mutually exclusive fashion by age (65 years or less, greater than 65 years), gender, education level (+/- university degree), and context (colon cancer, rectal cancer, receipt of radiotherapy, presence of a stoma). Eligible patients must be at least 18 years of age, understand English, with confirmed colorectal cancer. Patients will be contacted by mail with an interview invitation and consent form. This will take place as soon after treatment as possible to minimize recall bias. Non-responders will be contacted by telephone to request participation.

Interviews will be conducted with 15 surgeons, medical oncologists, and radiation oncologists (total n = 45) who care for patients with colorectal cancer and are affiliated with province based cancer centres. Surgeons, medical oncologists, and radiation oncologists will be identified through Cancer Care Ontario, the provincial cancer agency. Physicians will be selected that vary in non-mutually exclusive fashion by age (<50 years, >50 years), gender, years in practice (≤ 5 years, 6 to 10 years, >10 year) and geographic location (academic, community centre). Eligible physicians will be contacted by mail with an interview invitation and consent form. Non-responders will be contacted by telephone to request participation.

We will not seek interviews with physician-patient dyads because we are not measuring concordance between patient preference and actual receipt of shared decision making. Hence, even if physicians decline to participate we will seek interviews with their patients, and vice versa. To encourage participation, we will use strategies to increase survey response rates, including a hand signed, personalized cover letter on institutional letterhead and a pre-addressed, stamped return envelope [26,27].

### Data collection

Semi-structured interviews will be conducted, and all interviews will be audio-recorded and transcribed by an external professional. A single individual will conduct all of the interviews to enhance validity by maintaining internal consistency.

The main objectives of the patient interviews are to explore: patients' perceptions of the information they received and were asked to provide during treatment decision making; patients' perceptions of the extent to which they participated in treatment decision making and their preferred level of participation (role-matching); how satisfied patients were with their involvement in treatment decision making; and patients' perceptions on how the treatment decision-making process could be improved. The main objectives of the physician interviews are to explore: physicians' perceptions of what shared decision making is; the extent to which physicians use and/or facilitate shared decision making; physicians' perceptions of the usefulness of shared decision making; and strategies used by physicians to incorporate shared decision making into their clinical practice. Prior to the beginning of the study, the patient and physician interviews will be pilot tested on a small number of patients and physicians to refine wording and flow of questions. Interview guides for the patients and physicians interviews will be based on the conceptual framework developed for this study.

### Data analysis

Standard principles of qualitative research will be used to sample patients and physicians representing various characteristics, contexts, and settings. Hence, sampling will be purposive to select individuals whose opinions may vary according to these attributes. In qualitative research detailed information from a representative rather than a large number of cases is needed. Sample size is capped when no further unique themes emerge from successive interviews (informational redundancy) [28]. This is determined at the time of the data analysis that is concurrent with the data collection. If information redundancy is not achieved in patient or physician subcategories, additional interviews will be conducted.

An inductive, grounded approach will be used for qualitative analysis of interview transcripts using constant comparative analysis. This means that themes will be allowed to emerge from the collected data, and progress through three defined processes: description, categorical/conceptual ordering, and theorizing [28-31]. This involves repeated reading of transcripts, development of a coding scheme reflecting unique ideas, application of the coding scheme to transcript text, and grouping of coded text by theme. Consistent with constant comparative analysis, open and axial coding of interview transcripts will occur simultaneously, as data collection and analysis are concurrent [30,31]. Open coding recognizes ideas or concepts identified by study participants by analyzing transcripts line-by-line in their entirety, and then groups concepts together to form categories and subcategories, often using participant's own words as code names to ensure groundedness [30,31]. In this initial stage of constant comparative analysis, data is coded in every way possible to uncover all ideas.

Next, axial coding will be used to make connections between categories and subcategories of codes. Codes generated from open coding will be collapsed and grouped into mutually exclusive categories focusing on three interrelated aspects of Strauss and Corbin's (1990) coding paradigm: individual actions or behaviours, situational context, and consequences of the behaviours [28]. Repeating ideas will be assembled into themes based on content similarity. A theme is an implicit topic that organizes a group of repeating ideas. Themes will be similarly reviewed and assembled into abstract theoretical constructs based on their relation to one another and their ability to explain factors influencing shared decision-making preferences and behaviours. Theoretical constructs organize themes into larger, more abstract ideas. Themes and theoretical constructs will be tabulated to compare barriers, enablers, and outcomes of shared decision making by patient, physician, and contextual factors. Finally theoretical constructs will be organized into a theoretical narrative, which summarizes what we have learned and bridges the research objectives with participants' subjective experience.

To improve the reliability of these findings, two investigators will individually analyze and code all transcripts. They will meet to compare findings and achieve consensus through discussion. Collaborative coding by multiple individuals minimizes the chance that important thematic ideas are overlooked, and ensures that the organization of the data and the resulting conceptual theory is transparent [28].

## Discussion

The results of this study will provide better understanding of the current use and enablers and barriers to the use of shared decision making from both the patient and physician perspective in the setting of colorectal cancer. This study is the first to examine both patient and physician perspectives on the use of shared decision making for colorectal cancer in Canada or elsewhere. Fundamentally, it is an important study because it will provide a framework that can be used to describe the shared decision-making process and to make recommendations about how to best facilitate this process. The development of a framework is the critical first step necessary to explore the information and decision support needs of colorectal cancer patients.

## Competing interests

The authors declare that they have no competing interests.

## Authors' contributions

All authors participated in the design of the study. AG and EDK developed the qualitative research methods. EDK conducted the interviews and was responsible for the overall execution of the study. EDK, GS, SS, and MLC reviewed the interview transcripts that have been conducted. AG led qualitative data analysis methods. DU assisted with interpretation of synthesized data analyses. All authors read and approved the final manuscript.
